# Supplementary material for: Multi-omics integration analysis identifies novel genes for alcoholism with potential overlap with neurodegenerative diseases
Source: Nat Commun. 2021 Aug 20;12:5071. doi: 10.1038/s41467-021-25392-y (PMC8379159; doi:10.1038/s41467-021-25392-y)
Supplement: Supplementary file 1 — Supplementary Information [file 41467_2021_25392_MOESM1_ESM.pdf]

Supplementary Figures for manuscript, “Multi-omics integration analysis identifies novel genes for alcoholism with potential link to neurodegenerative diseases”.

Figures 1-6 are generated using AUD meta-analysis summary statistics through FUMA (https://fuma.ctglab.nl).

Figures 7-15 represents the Manhattan plot for individual SMR analyses presented in the manuscript.

Figure 16 shows the results of disease enrichment analysis using SMR summary statistics of DPW GWAS.

Supplementary Figure 1: Manhattan plot for AUD GWAS meta-analysis. X-axis of the plot represents each chromosome and y-axis represents the -log10 (P) values of each SNP at this locus. P-values for mQTL meta-analysis were obtained using conventional inverse-variance-weighted meta-analysis. (Figure generated through FUMA)

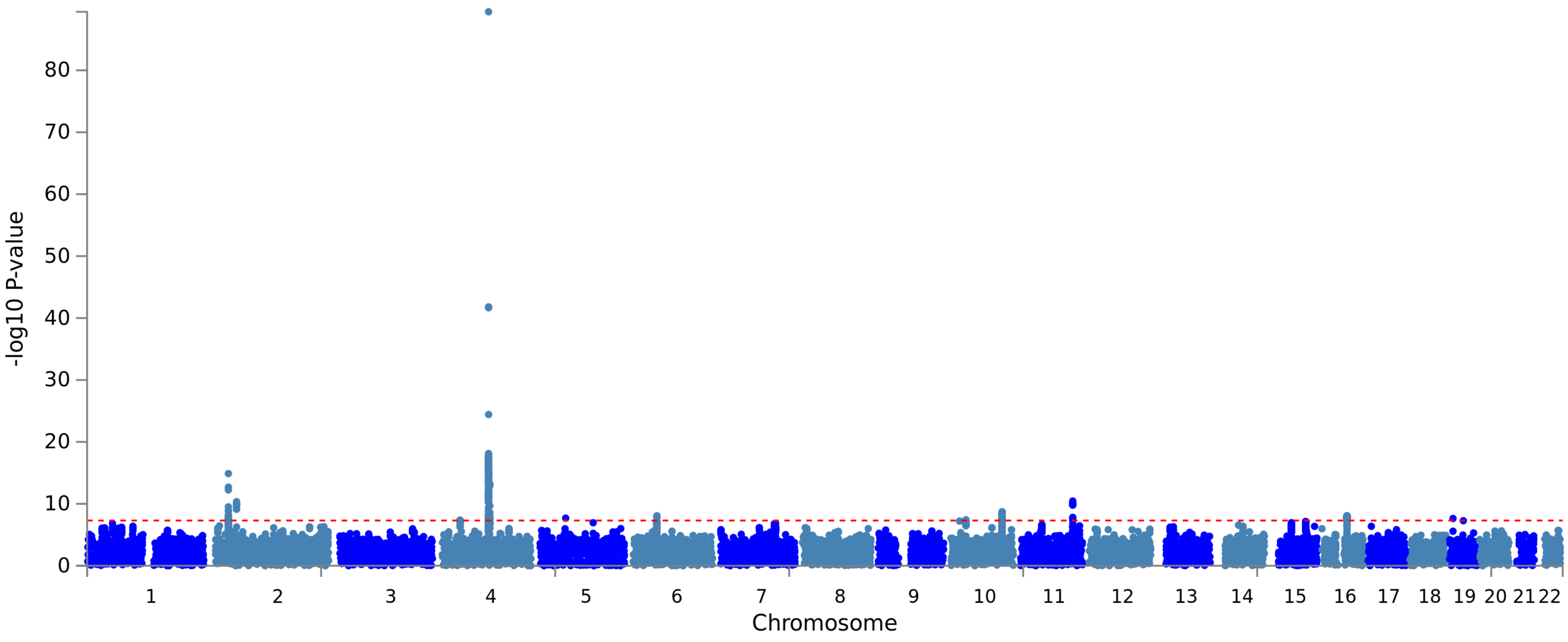

Supplementary Figure 2: Manhattan plot for gene-based association analysis results from MAGMA for AUD GWAS meta-analysis. X-axis of the plot represents each chromosome and y-axis represents the -log10 (P) values of each gene. P-values shown in this plot resulted from MAGMA analysis using default settings on FUMA webpage. (Figure generated through Fuma)

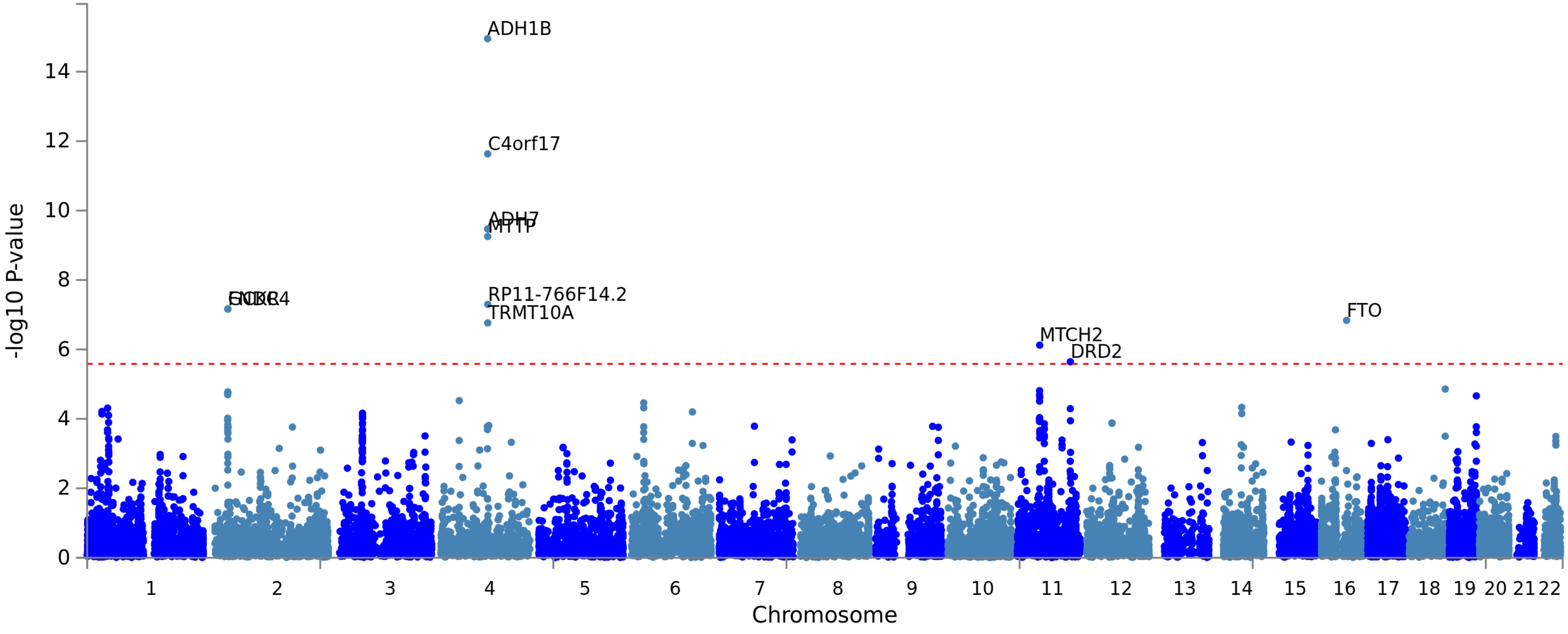

Supplementary Figure 3: Distribution of AUD GWAS SNPs across the genome. This histogram displays the proportion of SNPs which have corresponding functional annotations assigned by ANNOVAR. Bars are colored by -log2 (enrichment) relative to all SNPs in the selected reference panel. Fisher's exact test (two side) is performed for each annotation to calculate the P-values. (Annotations and information from FUMA)

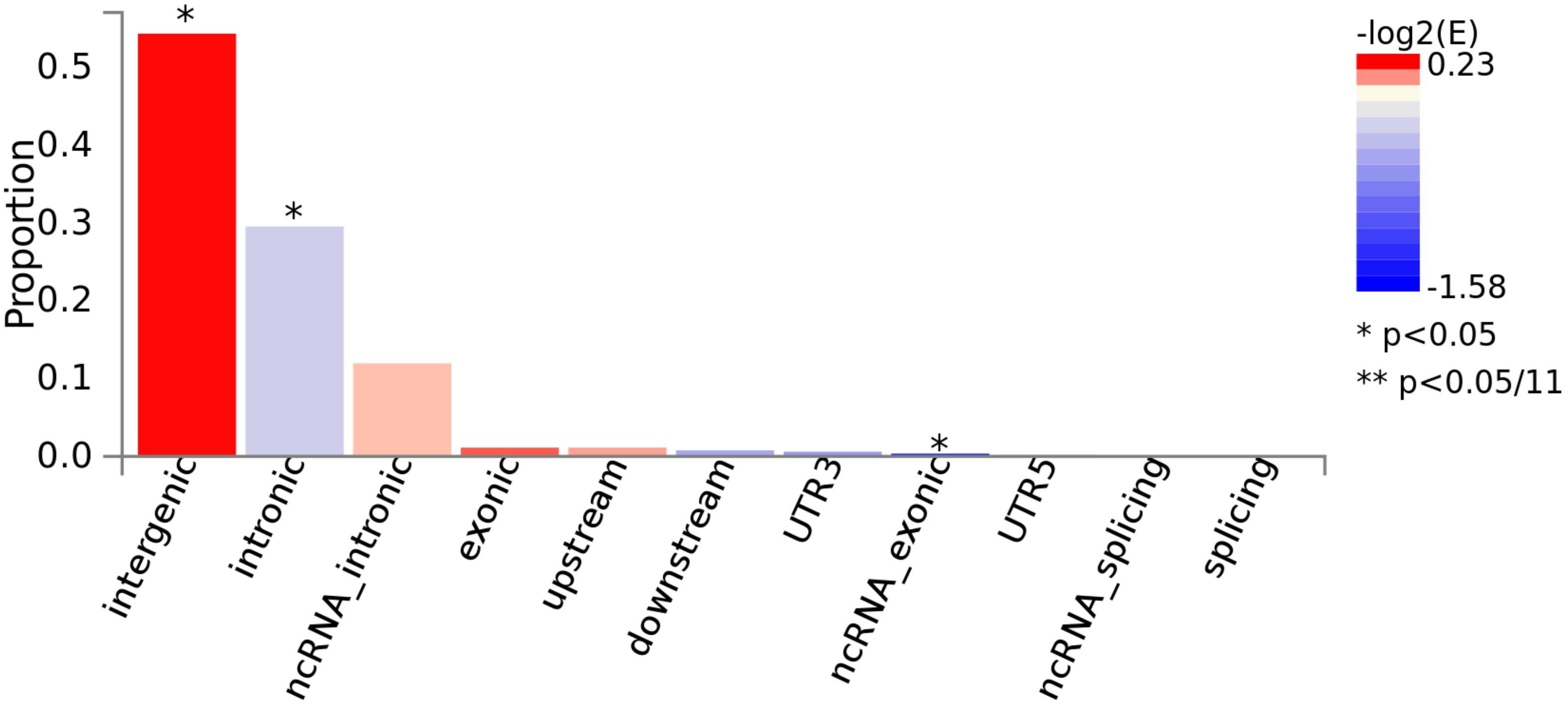

Supplementary Figure 4: Number of SNPs and Genes at each locus associated with AUD. This histogram represents the summary results for AUD meta-analysis per genomic locus. Each genomic locus can have more than one independent SNP. (Annotations and information from FUMA)

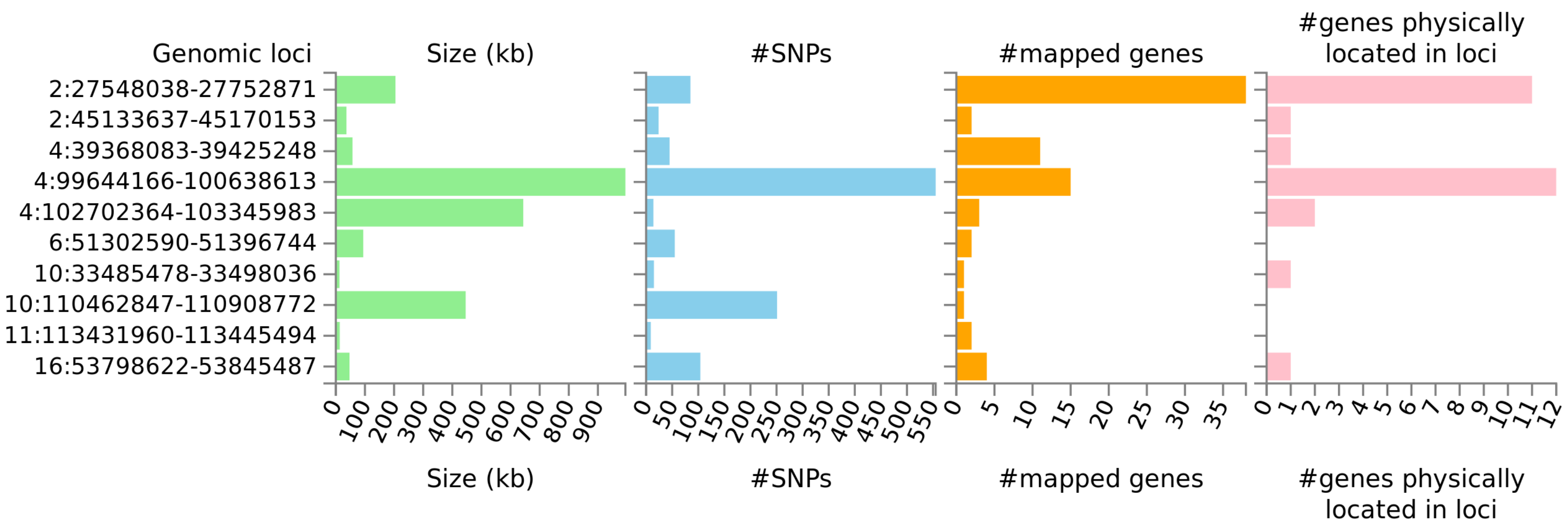

Supplementary Figure 5: Number of SNPs and Genes at each locus common between AUD and DPW GWASs. This histogram represents the summary results for common SNPs between AUD meta-analysis and DPW meta-analysis per genomic locus. Each genomic locus can have more than one independent SNP.
(Annotations and information from FUMA)

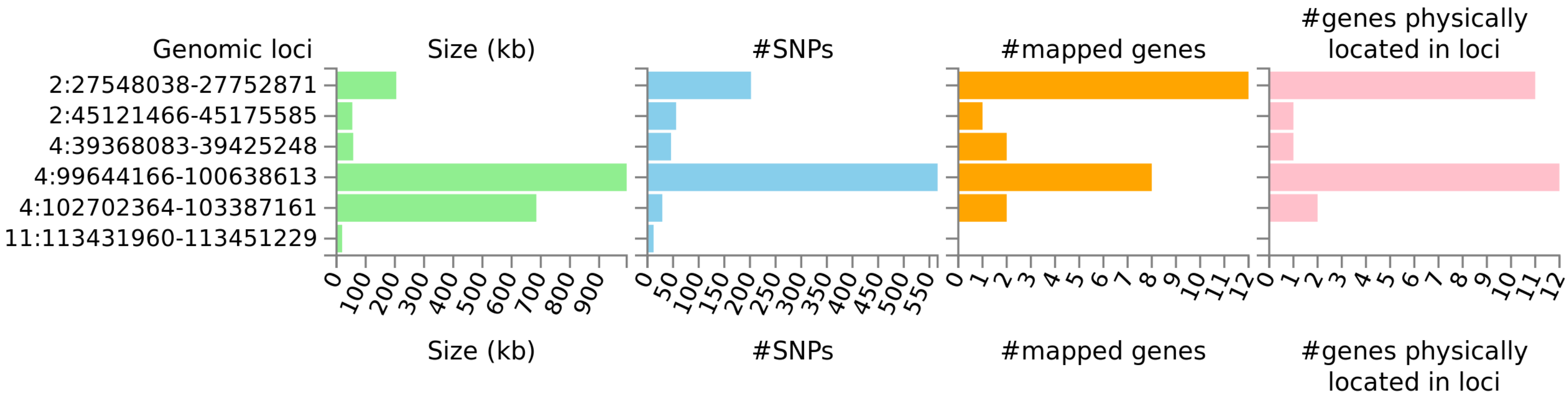

Supplementary Figure 6: Distribution of SNPs shared between AUD and DPW GWASs across the genome. This histogram displays the proportion of SNPs which have corresponding functional annotations assigned by ANNOVAR. Bars are colored by -log2 (enrichment) relative to all SNPs in the selected reference panel. Fisher's exact test (two side) is performed for each annotation to calculate the P-values. (Annotations and information from FUMA)

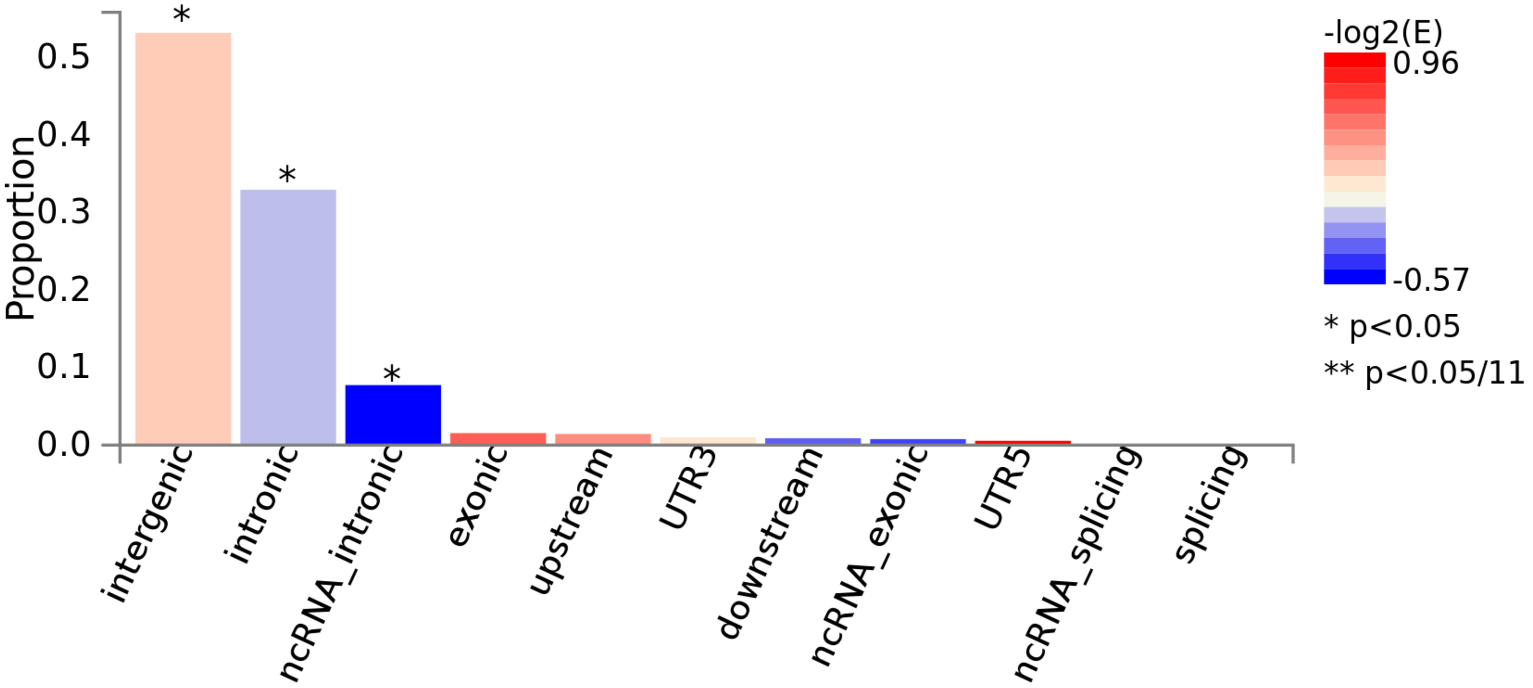

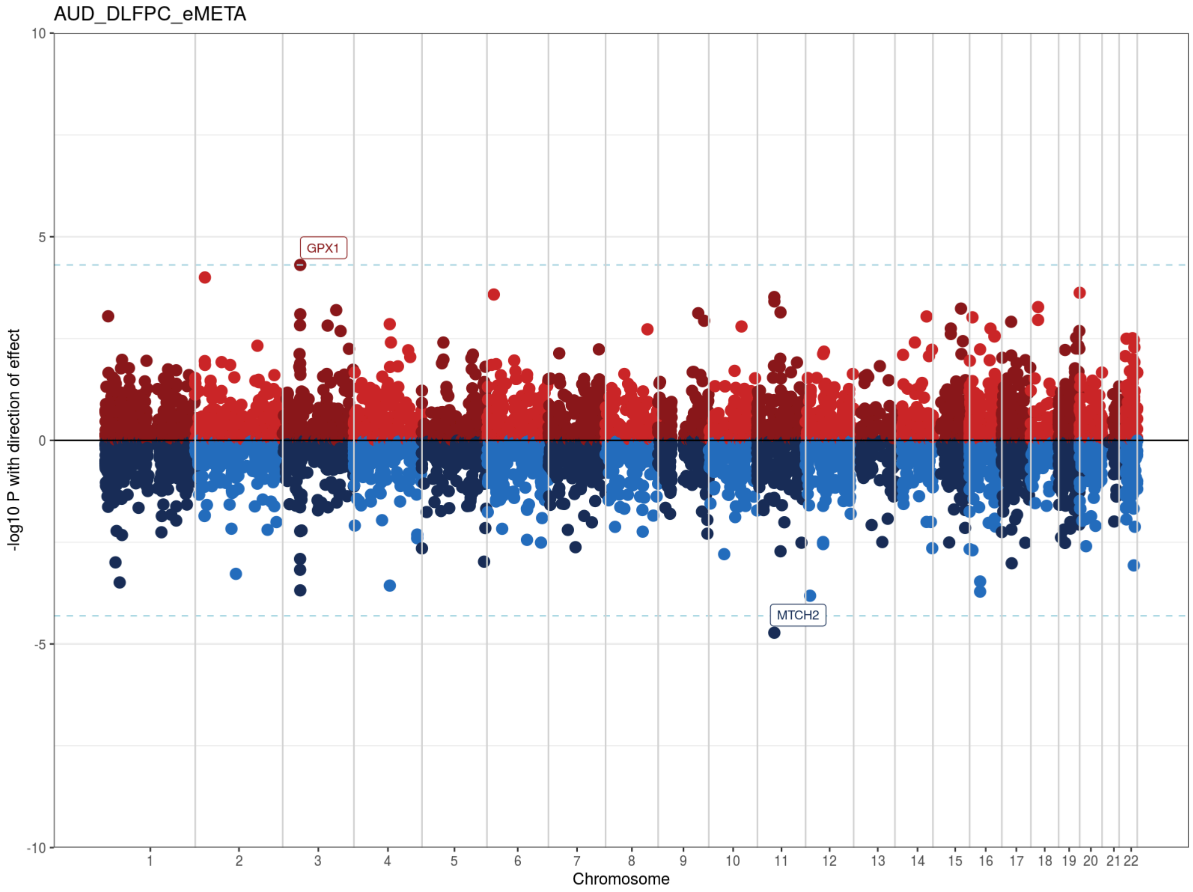

Supplementary Figure 7: Results of SMR based integration analysis of AUD GWAS with eQTL from adult brain. X-axis represents the chromosomes and Y axis shows the -log 10 (P) from SMR analysis with the direction of effect (Beta values from SMR analysis). Dotted line on each side represents the -log 10 (P) at 20% FDR. Genes marked on the plots represent the genes nominated through threshold of co-localization defined by False discovery rate of 20 % (FDR < 20%).

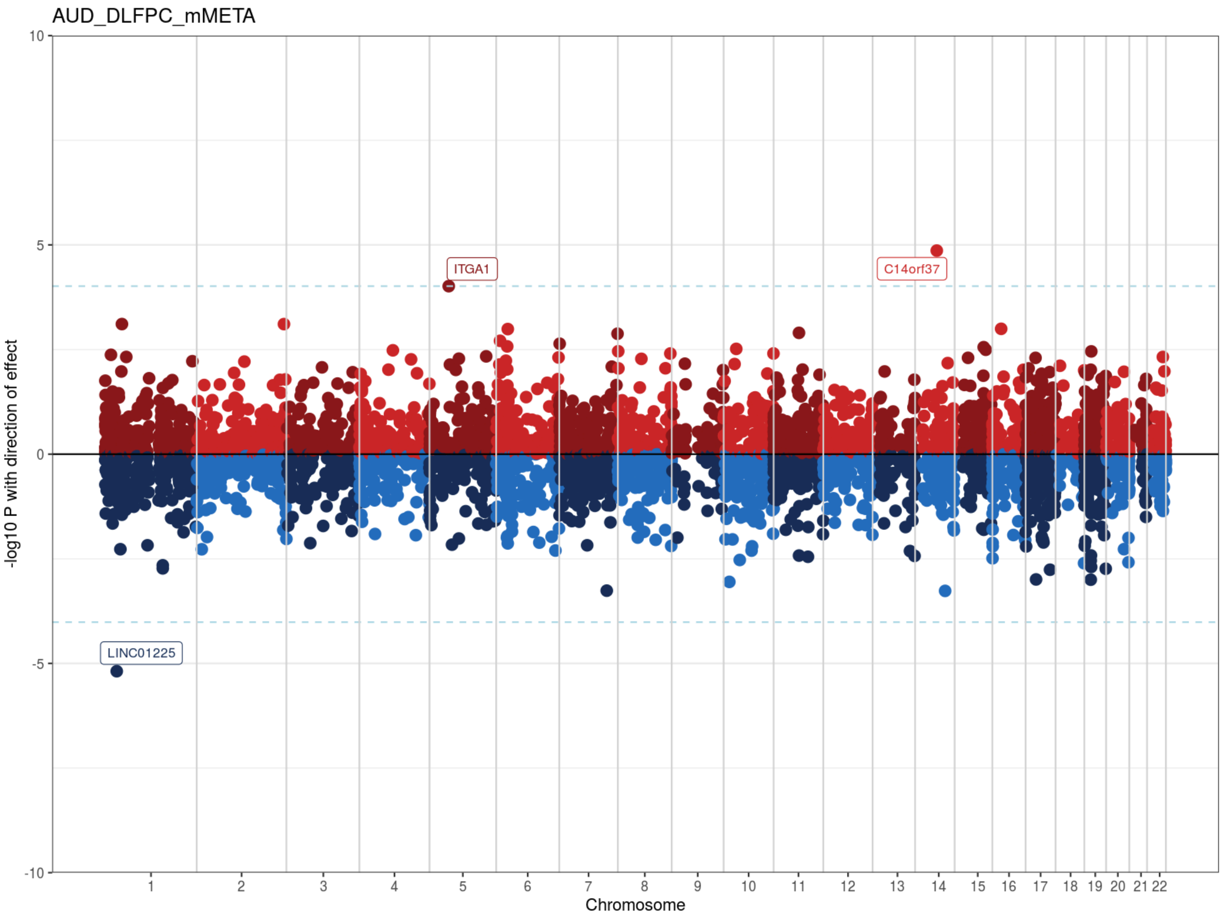

Supplementary Figure 8: Results of SMR based integration analysis of AUD GWAS with mQTL from adult brain. X-axis represents the chromosomes and Y axis shows the -log 10 (P) from SMR analysis with the direction of effect (Beta values from SMR analysis). Dotted line on each side represents the -log 10 (P) at 20% FDR. Genes marked on the plots represent the genes nominated through threshold of co-localization defined by False discovery rate of 20 % (FDR < 20%).

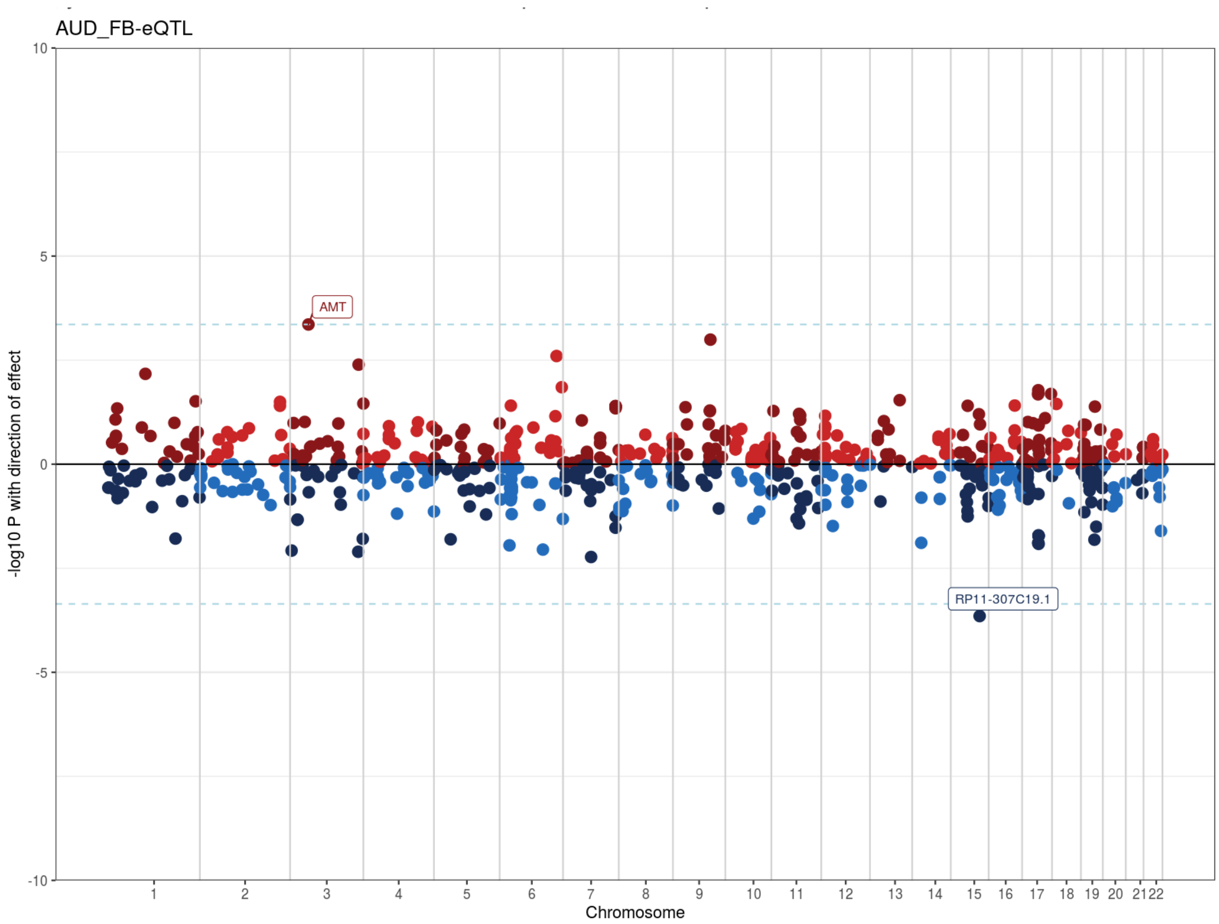

Supplementary Figure 9: Results of SMR based integration analysis of AUD GWAS with eQTL from fetal brain. X-axis represents the chromosomes and Y axis shows the -log 10 (P) from SMR analysis with the direction of effect (Beta values from SMR analysis). Dotted line on each side represents the -log 10 (P) at 20% FDR. Genes marked on the plots represent the genes nominated through threshold of co-localization defined by False discovery rate of 20 % (FDR < 20%).

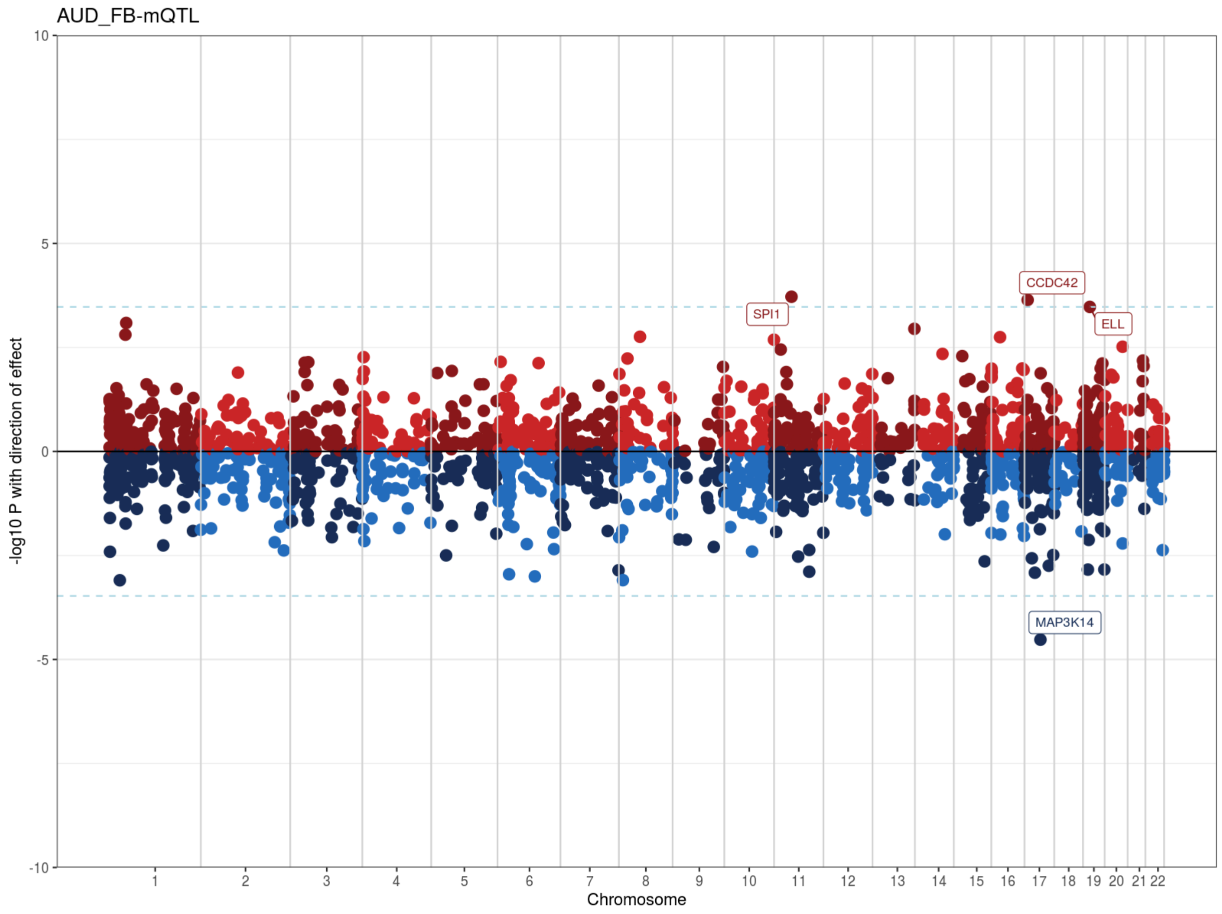

Supplementary Figure 10: Results of SMR based integration analysis of AUD GWAS with mQTL from fetal brain. X-axis represents the chromosomes and Y axis shows the -log 10 (P) from SMR analysis with the direction of effect (Beta values from SMR analysis). Dotted line on each side represents the -log 10 (P) at 20% FDR. Genes marked on the plots represent the genes nominated through threshold of co-localization defined by False discovery rate of 20 % (FDR < 20%).

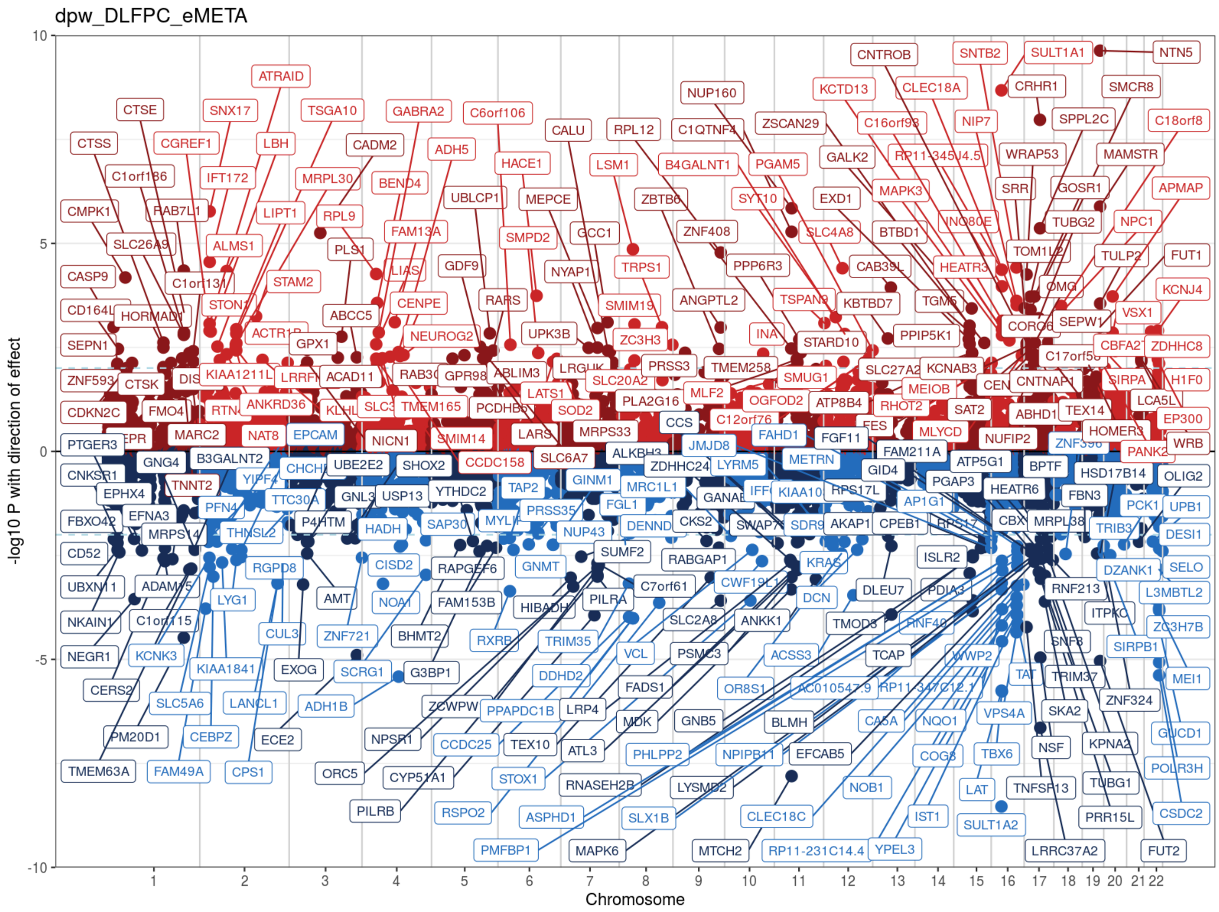

Supplementary Figure 11: Results of SMR based integration analysis of DPW GWAS with eQTL from adult brain. X-axis represents the chromosomes and Y axis shows the -log 10 (P) from SMR analysis with the direction of effect (Beta values from SMR analysis). Dotted line on each side represents the -log 10 (P) at 20% FDR. Genes marked on the plots represent the genes nominated through threshold of co-localization defined by False discovery rate of 20 % (FDR < 20%).

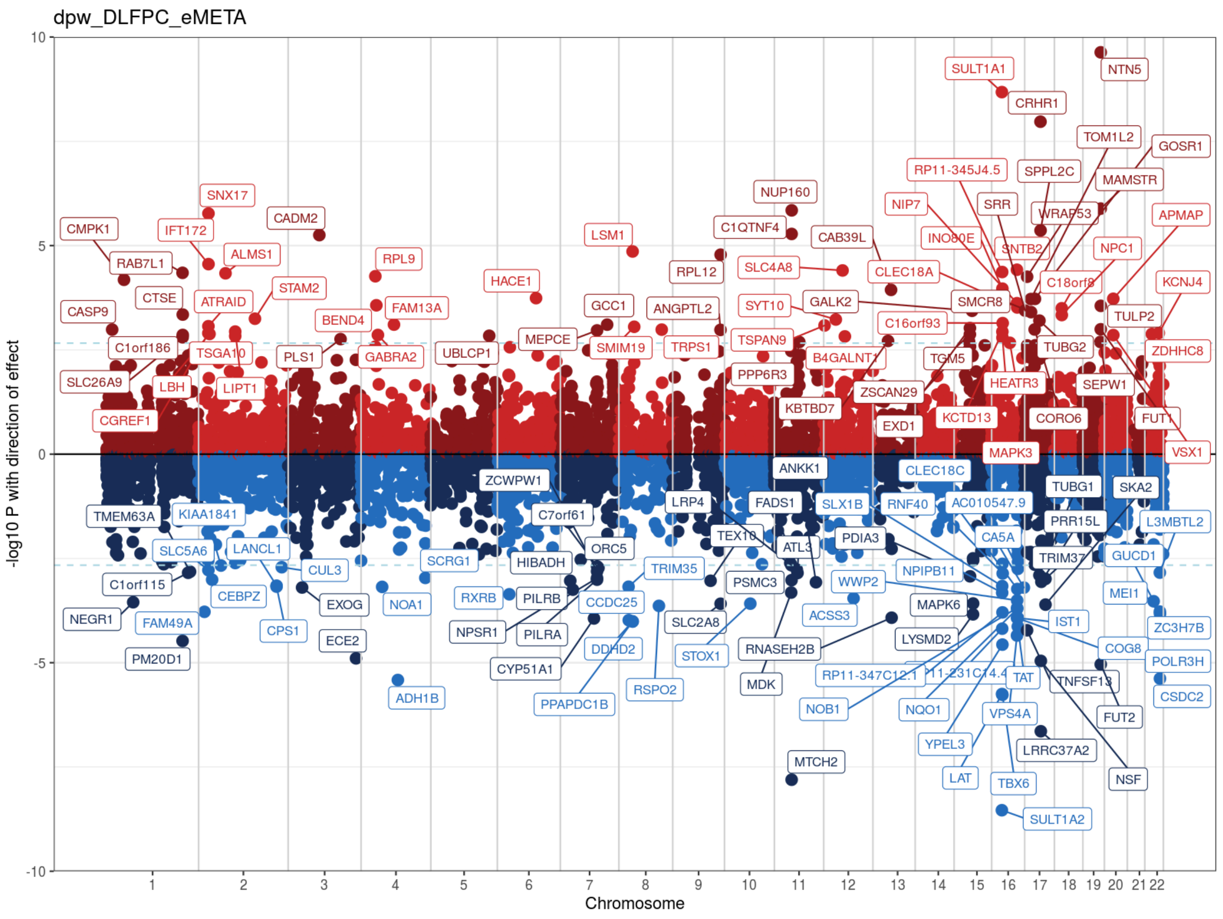

Supplementary Figure 12: Results of SMR based integration analysis of DPW GWAS with eQTL from adult brain. X-axis represents the chromosomes and Y axis shows the -log 10 (P) from SMR analysis with the direction of effect (Beta values from SMR analysis). Dotted line on each side represents the -log 10 (P) at 20% FDR. Genes marked on the plots represent the genes nominated through threshold of co-localization defined by False discovery rate of 20 % (FDR < 20%).

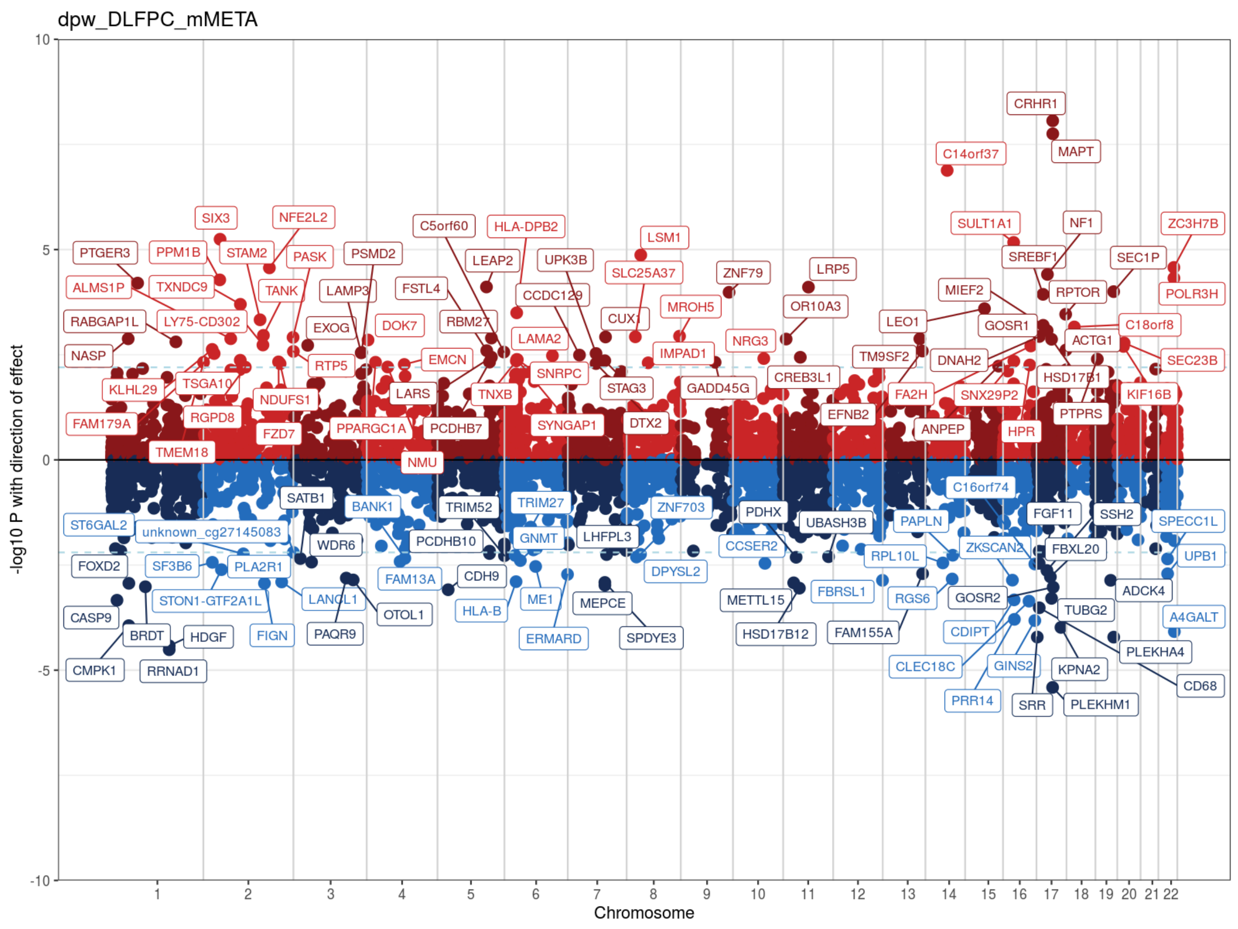

Supplementary Figure 13: Results of SMR based integration analysis of DPW GWAS with mQTL from adult brain. X-axis represents the chromosomes and Y axis shows the -log 10 (P) from SMR analysis with the direction of effect (Beta values from SMR analysis). Dotted line on each side represents the -log 10 (P) at 20% FDR. Genes marked on the plots represent the genes nominated through threshold of co-localization defined by False discovery rate of 20 % (FDR < 20%).

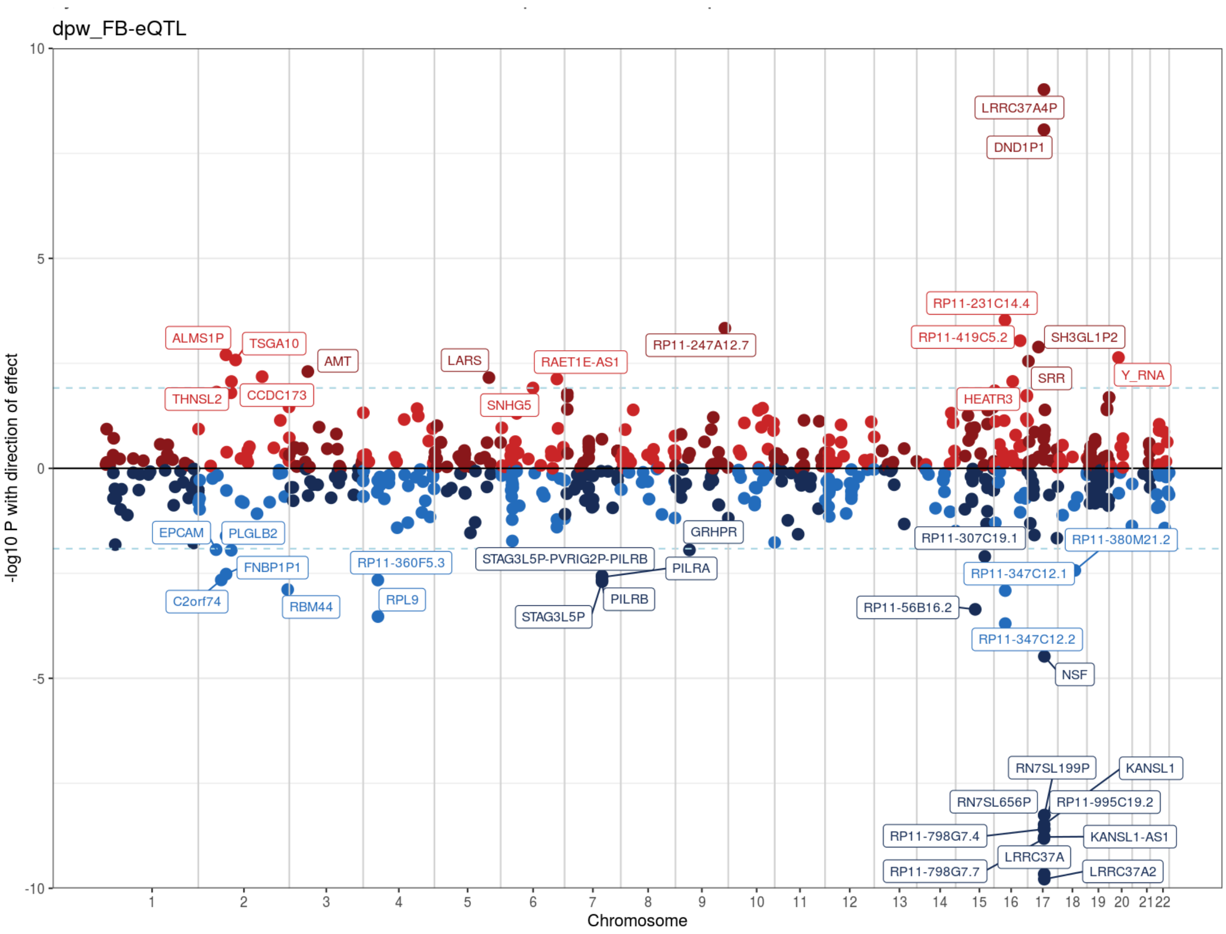

Supplementary Figure 14: Results of SMR based integration analysis of DPW GWAS with eQTL from fetal brain. X-axis represents the chromosomes and Y axis shows the -log 10 (P) from SMR analysis with the direction of effect (Beta values from SMR analysis). Dotted line on each side represents the -log 10 (P) at 20% FDR. Genes marked on the plots represent the genes nominated through threshold of co-localization defined by False discovery rate of 20 % (FDR < 20%).

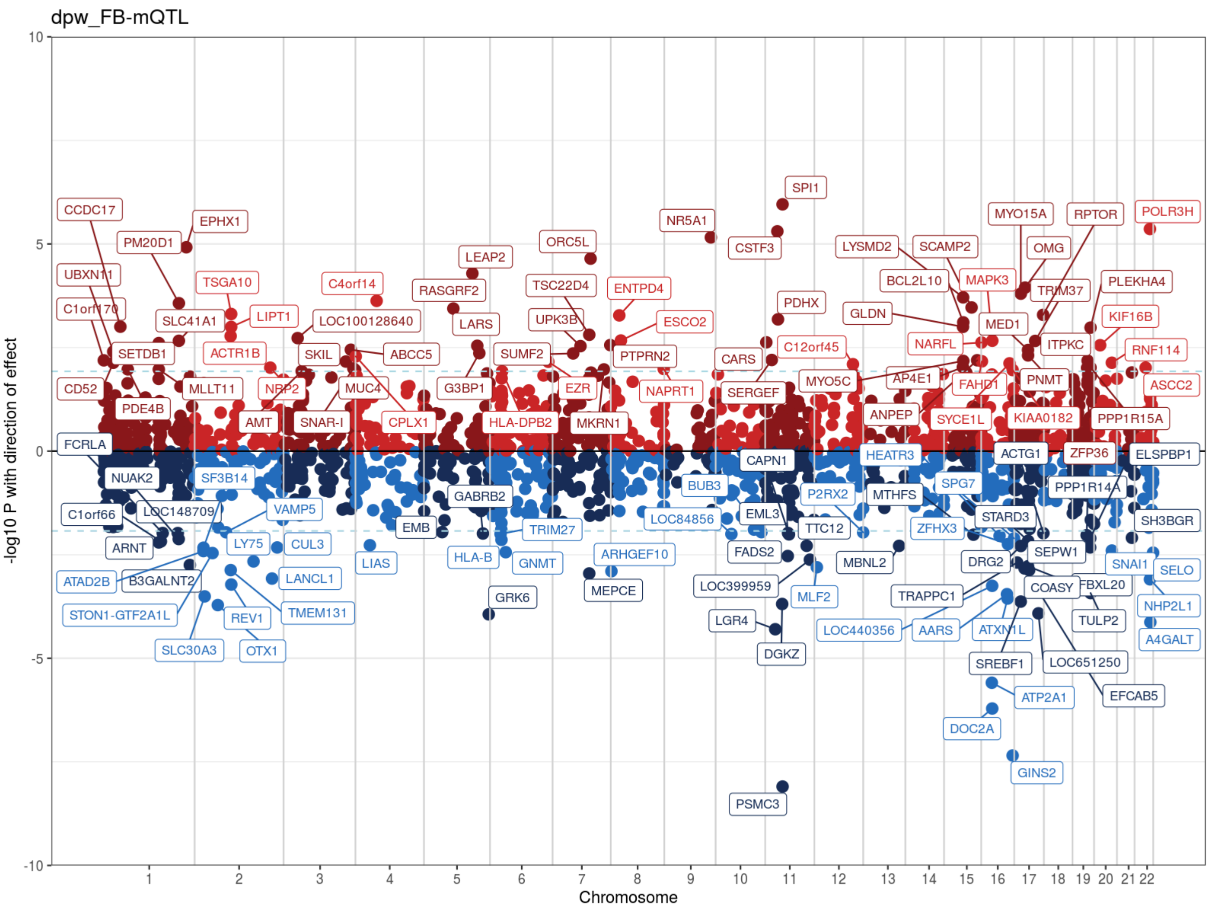

Supplementary Figure 15: Results of SMR based integration analysis of DPW GWAS with mQTL from fetal brain. X-axis represents the chromosomes and Y axis shows the -log 10 (P) from SMR analysis with the direction of effect (Beta values from SMR analysis). Dotted line on each side represents the -log 10 (P) at 20% FDR. Genes marked on the plots represent the genes nominated through threshold of co-localization defined by False discovery rate of 20 % (FDR < 20%).

Analysis: genes_sig_dpw_brain – 2020–03–27 11:36 AM

Supplementary Figure 16: This figure shows the results for disease enrichment analysis performed using IPA. IPA calculates the P values for enrichment using Fishers's Exact test (right tailed). The x-axis of this horizontal plot represents the disease and y-axis is the corresponding -log(P) value. P values presented here are not corrected for multiple test correction. The vertical line near -log P = 1.25 represents the threshold for nominal significance.

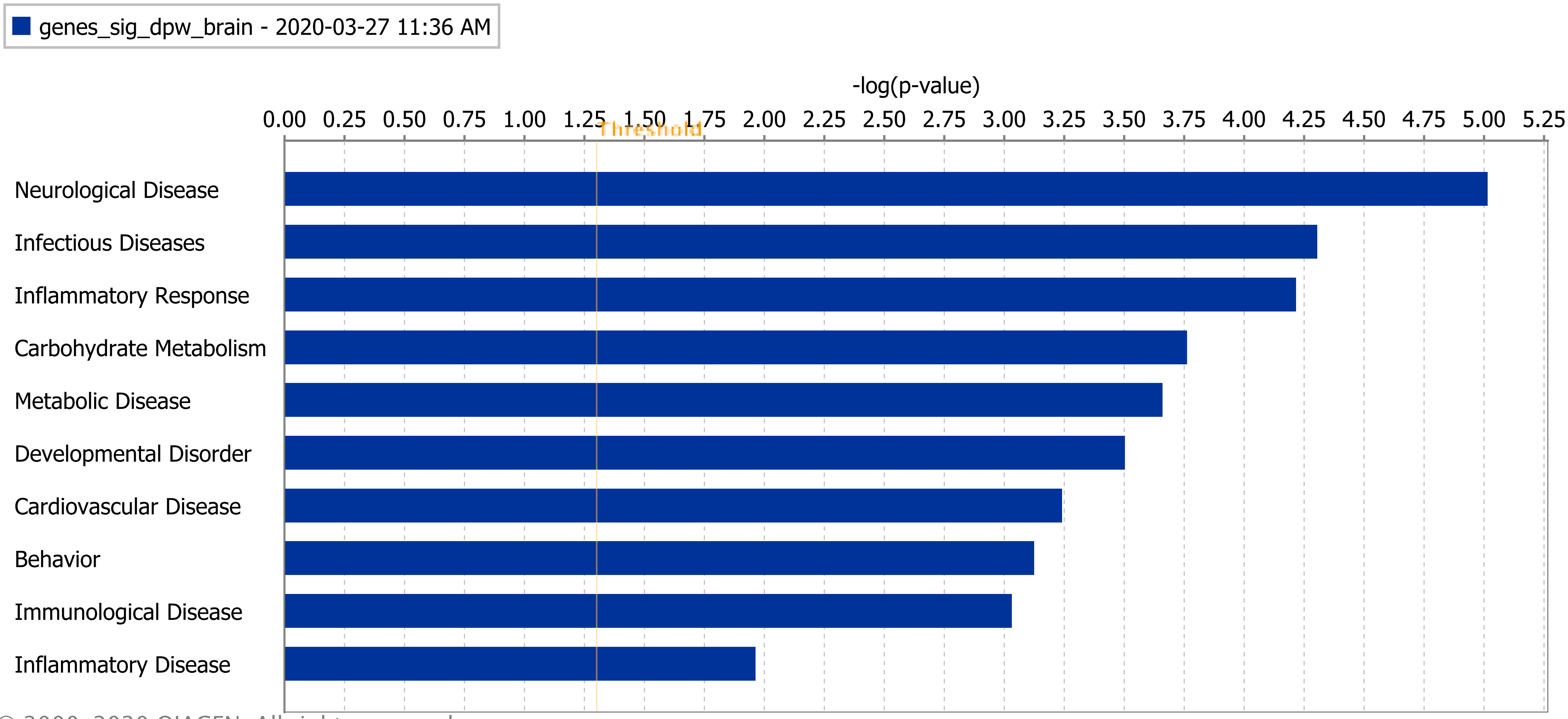

© 2000–2020 QIAGEN. All rights reserved.
