# Supplementary material for: Multi-omics integration analysis identifies novel genes for alcoholism with potential overlap with neurodegenerative diseases
Source: Nat Commun. 2021 Aug 20;12:5071. doi: 10.1038/s41467-021-25392-y (PMC8379159; doi:10.1038/s41467-021-25392-y)
Supplement: Supplementary file 3 — Description of Additional Supplementary Files [file 41467_2021_25392_MOESM3_ESM.pdf]

Description of Additional Supplementary files

Title: Supplementary Data 1
Description: Brain eQTL and mQTL datasets used for SMR analysis

Title: Supplementary Data 2
Description: LDSC analysis of AUD GWAS with multi-tissue chromatin marks

Title: Supplementary Data 3
Description: LDSC analysis of DPW GWAS with multi-tissue chromatin marks

Title: Supplementary Data 4
Description: SMR analysis results with summary statistics of AUD GWAS (No GWAS P value threshold)

Title: Supplementary Data 5
Description: SMR analysis results with summary statistics of DPW GWAS (With GWAS P value threshold)

Title: Supplementary Data 6
Description: SMR analysis results with summary statistics of DPW GWAS (No GWAS P value threshold)

Title: Supplementary Data 7
Description: AUD genes that also replicated in DPW SMR analysis

Title: Supplementary Data 8
Description: Results of pathway analysis with genes prioritized in DPW integration analysis

Title: Supplementary Data 9
Description: SMR analysis using summary statistics of AUD and DPW meta-analysis using MTAG
