# Supplementary material for: Multi-omics integration analysis identifies novel genes for alcoholism with potential overlap with neurodegenerative diseases
Source: Nat Commun. 2021 Aug 20;12:5071. doi: 10.1038/s41467-021-25392-y (PMC8379159; doi:10.1038/s41467-021-25392-y)
Supplement: Supplementary file 13 — Reporting Summary [file 41467_2021_25392_MOESM13_ESM.pdf]

nature portfolio

Corresponding author(s): Manav Kapoor

Last updated by author(s): Aug 2, 2021

Reporting Summary

Nature Portfolio wishes to improve the reproducibility of the work that we publish. This form provides structure for consistency and transparency in reporting. For further information on Nature Portfolio policies, see our Editorial Policies and the Editorial Policy Checklist.

Statistics

For all statistical analyses, confirm that the following items are present in the figure legend, table legend, main text, or Methods section.

n/a	Confirmed	
☐	☒	The exact sample size (*n*) for each experimental group/condition, given as a discrete number and unit of measurement
☒	☐	A statement on whether measurements were taken from distinct samples or whether the same sample was measured repeatedly
☐	☒	The statistical test(s) used AND whether they are one- or two-sided<br>*Only common tests should be described solely by name; describe more complex techniques in the Methods section.*
☐	☒	A description of all covariates tested
☐	☒	A description of any assumptions or corrections, such as tests of normality and adjustment for multiple comparisons
☒	☐	A full description of the statistical parameters including central tendency (e.g. means) or other basic estimates (e.g. regression coefficient) AND variation (e.g. standard deviation) or associated estimates of uncertainty (e.g. confidence intervals)
☒	☐	For null hypothesis testing, the test statistic (e.g. *F*, *t*, *r*) with confidence intervals, effect sizes, degrees of freedom and *P* value noted<br>*Give P values as exact values whenever suitable.*
☒	☐	For Bayesian analysis, information on the choice of priors and Markov chain Monte Carlo settings
☒	☐	For hierarchical and complex designs, identification of the appropriate level for tests and full reporting of outcomes
☒	☐	Estimates of effect sizes (e.g. Cohen's *d*, Pearson's *r*), indicating how they were calculated

Our web collection on statistics for biologists contains articles on many of the points above.

Software and code

Policy information about availability of computer code

Data collection	For the work presented in this manuscript, no new data was collected. We used already available data to perform meta-analyses and integrative multi-omic analyses.
Data analysis	LDSC (v1.0.1)<br>SMR (version 1.03)<br>GCTA (1.93.0beta)<br>UCSC browser (https://genome.ucsc.edu)<br>Pipelines used in the analysis can be accessed at the GitHub repository (https://github.com/kapoormanav/alc_multiomics) [DOI: 10.5281/zenodo.5076223.

For manuscripts utilizing custom algorithms or software that are central to the research but not yet described in published literature, software must be made available to editors and reviewers. We strongly encourage code deposition in a community repository (e.g. GitHub). See the Nature Portfolio guidelines for submitting code & software for further information.

Data

Policy information about availability of data

All manuscripts must include a data availability statement. This statement should provide the following information, where applicable:

- Accession codes, unique identifiers, or web links for publicly available datasets
- A description of any restrictions on data availability
- For clinical datasets or third party data, please ensure that the statement adheres to our policy

The individual Manhattan plots with SMR analysis for all conditions can be found in supplementary information figures 7-15. Supplementary figures 1-4 reports the

AUD-meta association plots generated through FUMA. Additionally all the results can be visualized at our Shiny web app (https://lcad.shinyapps.io/alc_multiomics/).
No raw data was generated in this manuscript. AUD meta-analysis summary statistics along with summary statistics of all SMR analyses are available to download at figshare.com:
AUD meta-analysis summary statistics: 10.6084/m9.figshare.15054198.v1
SMR Input BESD files for brain meta-analysis: 10.6084/m9.figshare.15054183.v1
mRNA expression analysis of alcohol consumption (corrected for BMI, AUDIT scores, age, sex and PMI): 10.6084/m9.figshare.15054171.v1
SMR results (Complete summary statistics files): 10.6084/m9.figshare.15054120

Field-specific reporting

Please select the one below that is the best fit for your research. If you are not sure, read the appropriate sections before making your selection.

- [x] Life sciences
- [ ] Behavioural & social sciences
- [ ] Ecological, evolutionary & environmental sciences

For a reference copy of the document with all sections, see nature.com/documents/nr-reporting-summary-flat.pdf

Life sciences study design

All studies must disclose on these points even when the disclosure is negative.

Sample size	This study used the summary statistics from largest available GWAS summary data for AUD to perform the meta-analysis. No new data was collected for the analyses.
Data exclusions	No data was excluded from the meta-analyses
Replication	Results of multi-omics integration based predicted mRNA expression analyses were replicated using measured mRNA expression in brain of alcoholics and controls.
Randomization	Mendelian Randomization based integration analyses were used in the current study
Blinding	NA (only summary statistics were used. No raw data was used in the study)

Reporting for specific materials, systems and methods

We require information from authors about some types of materials, experimental systems and methods used in many studies. Here, indicate whether each material, system or method listed is relevant to your study. If you are not sure if a list item applies to your research, read the appropriate section before selecting a response.

Materials & experimental systems

n/a	Involved in the study	
x		Antibodies
x		Eukaryotic cell lines
x		Palaeontology and archaeology
x		Animals and other organisms
	x	Human research participants
x		Clinical data
x		Dual use research of concern

Methods

n/a	Involved in the study	
x		ChIP-seq
x		Flow cytometry
x		MRI-based neuroimaging

Human research participants

Policy information about studies involving human research participants

Population characteristics	Analyses presented in this paper were performed on European American population. Data from other ethnicities was not used due to lack of appropriate sample size for eQTL/ mQTL datasets from other ethnicities.
Recruitment	NA
Ethics oversight	Icahn School of Medicine at Mount Sinai

Note that full information on the approval of the study protocol must also be provided in the manuscript.
