# Supplementary material for: Multi-omics integration analysis identifies novel genes for alcoholism with potential overlap with neurodegenerative diseases
Source: Nat Commun. 2021 Aug 20;12:5071. doi: 10.1038/s41467-021-25392-y (PMC8379159; doi:10.1038/s41467-021-25392-y)
Supplement: Supplementary file 2 — Peer Review File [file 41467_2021_25392_MOESM2_ESM.pdf]

nature portfolio

Peer Review File

Multi-omics integration analysis identifies novel genes for alcoholism with potential overlap with neurodegenerative diseases

Open Access This file is licensed under a Creative Commons Attribution 4.0 International License, which permits use, sharing, adaptation, distribution and reproduction in any medium or format, as long as you give appropriate credit to the original author(s) and the source, provide a link to the Creative Commons license, and indicate if changes were made. In the cases where the authors are anonymous, such as is the case for the reports of anonymous peer reviewers, author attribution should be to 'Anonymous Referee' followed by a clear attribution to the source work. The images or other third party material in this file are included in the article's Creative Commons license, unless indicated otherwise in a credit line to the material. If material is not included in the article's Creative Commons license and your intended use is not permitted by statutory regulation or exceeds the permitted use, you will need to obtain permission directly from the copyright holder. To view a copy of this license, visit http://creativecommons.org/licenses/by/4.0/.

Reviewers' Comments:

Reviewer #2:
Remarks to the Author:
Review of "Multi-omics integration analysis identifies novel genes for alcoholism with potential link to neurodegenerative diseases" by Kapoor et al.

The authors perform fine mapping of GWAS risk loci for alcohol use disorder (AUD) and drinks per week (DPW) using "multi-omics" approaches including Summary based Mendelian Randomization (SMR). SMR uses GWAS summary statistics and mQTL/eQTL data from fetal and adult brains to identify causal variants/genes. They identified several risk genes and fine-map/discuss in particular two loci; one on chromosome 11 (implicating SPI1 in both AUD and DPW) and one on chromosome 17 (implicating MAPT in DPW).
Additionally, the authors take their findings further and validate their SMR results for MAPT in expression data from post-mortem brain samples from individuals with AUD and controls.

This study represents a nice step towards further biological understanding of GWAS risk loci for AUD and DWP. Additionally, the authors have generated an online tool where readers can visualize the findings. I think this a great way to give readers the opportunity to look further into the results. I am positive about this study, however I still have some comments:

1) The authors use an FDR < 20% to claim significance in the SMR analyses. I could worry that this threshold is too liberal and additionally (at least to my understanding) the authors do provide any correction for the number tissues (adult or fetal brain) and type of regulatory variant (eQTL or mQTL).

2) In table 1a. and 1.b – what are the criteria for a gene to be included? Is it all genes with FDR < 20% in one of the analyzed tissues? – please add this information to the table legends and add information about what the "Diff_exp P value" represents.

3) The authors write "..mRNA expression of MAPT was associated with increased alcohol consumption (Figure 2c)" correct the text to (Figure 4c).

4) In the analysis testing for difference in expression of MAPT in postmortem brains of AUD cases and controls the authors report a significant association, however this is not significant after Bonferroni correction when correcting for the 61 genes reported in Table 2a. The results could also be validated using another bioinformatic method, such as doing sPrediXcan using models trained on brain tissues to test for association of gene expression with DPW.

5) In the pathway analyses – please give information about the number of pathways tested. I assume no pathways survive Bonferroni correction, instead the authors provide an FDR of 20%, and again I think this is very liberal in order to claim real significance. I would at least moderate the wording to state "suggestive significance" or something similar in the text.

6) At several places the authors write "AUD GWAS", I assume they refer to the AUD GWAS meta-analysis that they perform, but this is not clear. So please correct this to "AUD GWAS meta-analysis" throughout the text, when relevant.

7) In the introduction the authors write "DPW is genetically uncorrelated with most psychiatric disorders (except ADHD and tobacco use disorder) but correlated negatively with educational achievement and cardio-metabolic disease (which remains uncorrelated with PAU or AUD)". In the GSCAN paper DPW has a non-significant genetic correlation with educational attainment of rg=0.01 (including 23andMe individuals in the DPW sample), and Kranzler et al. found a positive genetic correlation between alcohol consumption and educational attainment. So could the authors check if the sentence instead should be "…but correlated positively with educational achievement".

Reviewer #3:
Remarks to the Author:
The focus of this work is to integrate -omics data (expression, methylation) with genome wide association study (GWAS) results for the two outcomes of alcohol use disorder (AUD) and drinks per week (DPW) to try to understand the functional significance of variants and determine causal candidate genes. It is novel in that it is the largest muli-omcs integration of these two outcomes, and will be of interest to the psychiatric and genetics research community. This is an impressive amount of work managing many different data sets and provides a candidate loci of interest. The main concerns were with the stated goals of a simultaneous approach for both outcomes, whether ancestry was addressed, and the presentation of the manuscript.

1. The introduction claims that this is an integrated approach and looks at the two phenotypes AUD and DPW "specifically as well as simultaneously." But it seems like most of the analyses examine AUD and DPW separately, then look later for overlap. For example the LDSC analysis seems to be completely separate, and not much discussion about overlap. Can the authors do more direct analysis (e.g. SNPs that intersect for the two outcomes) for a more integrative and simultaneous approach at later stages? Or they may need to downplay this description.

2. Ancestry is not discussed but there appears to be both European and African ancestry populations in the meta-analysis GWAS. But for the various e/mQTL meta-analysis performed by the authors and others, how similar are those populations to the populations used in the meta-analysis GWAS? Is ancestry adjusted for in any of the analyses? (GWAS, QTL, differential expression, etc)

3. Other concerns were about the presentation regarding enough detail for reproducibility and the statistical analysis. Since space is constrained, a supplemental materials/methods would help, in addition to improved organization of the figures/tables and supplemental figures/tables.

a. Although it may be obvious to some, it's still important to define all abbreviations (e.g, EUR, AFR, RIN). Also what is PMI? Is that supposed to be BMI?

b. What meta-analysis method is used fo the "eQTL meta-analysis in adult brain"? e.g., random-effects, etc. In general, for all of the meta-analyses, more detail would be important regarding the method, populations, sample sizes.

c. The numbering of the tables/figures are off. For example, Supplementary Table 1 seems to be 2, and Figures 2 and 3 seem to be 4 and 5 respectively. Other times Supplemental Figures and Tables are referred to in the text, but with no number.

d. There is a file of additional supplementary figures that are not described and did not appear to be referenced. They should not be included if not cited specifically.

e. The manuscript states "A large proportion (45%) of AUD and DPW associated SNPs were within intronic, UTR and non-coding regions of the genome." Is this percentage expected by chance?

f. Figure 6 seems unnecessary as a figure, the content can be displayed as a table or even in the text.

g. In the methods, please provide p-value or FDR cutoffs used in the different analyses. Is multiple testing adjustment performed for the differential expression analysis?

h. Can a similar figure relating MAPT gene expression to DPW as in Figure 4C, be performed for the

candidate gene SPI1 in Figure 5?

Reviewer #4:
Remarks to the Author:
This comprehensive analysis of existing human 'omics data relevant to the understanding of alcohol use disorder provides some replication of previously identified genes and evidence for novel associations. The manuscript is clearly written so it is easy to follow the different datasets and analyses conducted. The statistical methods applied are appropriate and rigorous. It represents a significant advancement for the field of alcohol genomics and should inspire basic scientists to prioritize some of these genes for further study.

Some questions and suggestions:

1) Title: It is not clear to me why a reference to neurodegenerative diseases merits mention in the title. It's not irrelevant, but I don't feel the results strongly point in this direction, or at least the way presented in the paper it is not emphasized.

2) Introduction, para 2: I do not understand why the authors refer to the Zhou et al paper (ref 10) as the "largest tranche of signals for any addictive disorder to date". In the very next sentence they refer to the Liu et al paper (ref 11) which included a larger sample and identified more loci/genes.

3) The emergence of SP11 and NUP160 as potentially important genes is interesting, especially given their high expression in myeloid cells. My colleagues who are experts in the connections between immunology and neurobiology have often questioned why we do not use perfusion for animal studies of mouse/rat brain for various RNA sequencing experiments. Their reasoning is that if you simply flash freeze the brains and use tissue from that, you will inevitably be assessing a lot of blood cells, not neuronal cells. Obviously, with human brains, perfusion is not possible. But this makes it difficult to interpret results where the genes involved may be more likely reflect changes in blood cells than neuronal cells. This is not to say such changes aren't important, but it seems some discussion is necessary to help the reader hypothesize how such changes may be functionally relevant to what is being studied in this context as a "brain" disease. This is more of a "big-picture" question, because as the authors point out in the discussion, other studies have identified an important role of immune networks in drinking behaviors (ref 25-31).

4) Minor point: Discussion, para 5 that begins, "We also identified other genes..." The word "also" occurs several times so the authors may wish to rework some of the writing.

5) I did not see any specific discussion about whether the authors attempted to identify possible sex differences for any of the analyses. It appeared to be included as a covariate for most or all, but in some cases it seems like they may have enough power to separate the sample by sex. Given the known sex differences in AUD, this has the potential to be very interesting and would add something new to the field. While the current paper is a fantastic effort and integration, it's really just adding more genes on top of other genes from previous 'omics studies, so a sex-specific analysis would add something new.

Along these lines, it would be useful for the authors to identify the genes in their tables that are new with this analysis and haven't emerged from previous publications. I don't say this to imply that I don't believe they've found anything new - I agree there is lots of great new stuff here. But it would really help the reader be able to distinguish which pieces are new and which genes now have lots of evidence for involvement in AUD.

6) I really like the use of ShinyApp to allow others to query the database and view the results.

However, it would be useful to add a section that clearly describes under a single heading where each of the datasets can be found, so that others could obtain the raw data for themselves.

7) This paper is limited to data collected from human studies. There would value in adding a paragraph or two in the discussion about genes identified in animal models. Unfortunately, I don't think a recent GeneWeaver analysis has been published for alcohol phenotypes, but I think Rohan Palmer's group has been working on it.

REVIEWER COMMENTS

We thank all three reviewers for their constructive critiques, that further enhance the quality of our manuscript. We have made several changes according to the reviewers' comments. A point-by-point response to the comments is provided below, with the resulting changes to the manuscript.

Reviewer #2 (Remarks to the Author):

This study represents a nice step towards further biological understanding of GWAS risk loci for AUD and DWP. Additionally, the authors have generated an online tool where readers can visualize the findings. I think this is a great way to give readers the opportunity to look further into the results. I am positive about this study, however I still have some comments:
We thank the reviewer for the positive statements.

1) The authors use an FDR < 20% to claim significance in the SMR analyses. I could worry that this threshold is too liberal and additionally (at least to my understanding) the authors do provide any correction for the number tissues (adult or fetal brain) and type of regulatory variant (eQTL or mQTL).

> *Ans: The SMR analysis is after we have already filtered our GWAS and eQTL summary statistics on strict P values thresholds, not the only threshold used. The reported genes in the integration analyses survived four different P value thresholds to be nominated as potential causal genes (GWAS P <= 5 x 10^{-5}; e/mQTL P <= 5 x 10^{-8}; Heidi P >= 0.05; SMR FDR <= 0.2). Thus the FDR values for SMR P are still conservative for identifying novel targets. Further, due to the complex LD structure at several loci many genes at a locus had similar correlated P values. Applying a more restrictive per gene FDR (without other threshold criteria) would have prioritized all genes at the few most significant loci, rather than prioritizing top candidate genes across the larger set of loci. For these reasons, we used restrictive thresholds for the initial steps and more liberal thresholds to report the final set of variants. We have noted this point under table 1 and 2. We have also noted above mentioned text in the result section.*

2) In table 1a. and 1.b what are the criteria for a gene to be included? Is it all genes with FDR < 20% in one of the analyzed tissues? Please add this information to the table legends and add information about what the Diff_exp P value represents.

> *Ans: Genes reported in the integration analyses survived four different P value thresholds to be nominated as potential causal genes (GWAS P <= 5 x 10^{-5}; e/mQTL P <= 5 x 10^{-8}; Heidi P >= 0.05; SMR FDR <= 0.2). This is now noted under each table.*

3) The authors write mRNA expression of MAPT was associated with increased alcohol consumption (Figure 2c), correct the text to (Figure 4c).

Ans: We have corrected the text.

4) In the analysis testing for difference in expression of MAPT in postmortem brains of AUD cases and controls the authors report a significant association, however this is not significant after Bonferroni correction when correcting for the 61 genes reported in Table

Ans: We agree that differential expression results will not survive multiple test correction. Although the differential expression results from brains of alcoholics and controls were generated on the largest dataset available to date (N = 138 total; alcohol consumption data for 92 brains), the small effect sizes of GWAS signals would require a much larger brain dataset to detect association of SNP mediated mRNA expression changes with the phenotype. For example, if we assume that SNP mediated expression is increasing the expression in alcoholics by 1.2 times (FC = 1.2; larger than most variants), then it requires data from at-least 200 brains (alcoholics + controls) to detect large number of association at FDR 0.05 (calculated using R package "ssizeRNA"). Despite this limitation our data prioritized key genes that were nominally associated, which is encouraging for further targeted studies. We have moderated the wording in the text that the association of MAPT in the independent dataset was replicated at a nominal significance level. We have also added the above-mentioned statement as a limitation of the study (Page 10; Lines 321-326).

2a. The results could also be validated using another bioinformatic method, such as doing sPrediXcan using models trained on brain tissues to test for association of gene expression with DPW.

We used the Transcriptome wide association analysis (TWAS) method to validate the results. Unlike prediXcan, TWAS uses multiple methods to predict the gene expression weights and outputs the results of the best prediction method. The TWAS analysis using the CommonMind eQTL dataset as a reference prioritized MAPT (TWAS P = 1.69×10^{-12}) as one of the strongest candidates in the 17q.21.31 locus. In comparison, the SMR p value for MAPT using our largest eQTL meta-analysis was slightly stronger (SMR P = 4.84×10^{-16}).

We also specifically want to point out that PrediXcan and TWAS have a small drawback. These methods require raw eQTL/ mQTL datasets to create the prediction models. Due to this caveat these methods are restricted to a smaller number of datasets for which the raw data or prediction weights are available (e.g. GTEx brain, CommonMind brains). Using the SMR method we were able to meta-analyze summary statistics from all large brain eQTL datasets and use it as a reference, boosting the power of our integration analyses. The TWAS results for MAPT locus are available as supplementary table 7 for comparison. The complete TWAS summary statistics will be available to download from the GitHub link.

5) In the pathway analyses please give information about the number of pathways tested. I assume no pathways survive Bonferroni correction, instead the authors provide an FDR of 20%,

and again I think this is very liberal in order to claim real significance. I would at least moderate the wording to state suggestive significance or something similar in the text.

Ans: In all, 410 pathways were tested using IPA and none of the pathway survived threshold for multiple test correction ($P_{Bonferroni} = 1.2 \times 10^{-4}$). Ingenuity Pathway Analysis of the prioritized genes associated with DPW showed suggestively significant enrichment for pathways related to TR (Thyroid hormone receptor)/RXR (Retinoic X receptor) activation ($P = 1.45 \times 10^{-4}$), and Lipoate biosynthesis (3.29×10^{-4}),_ which are very close to the threshold for Bonferroni correction.

We have also moderated the text to "suggestive significance" according to the reviewer's suggestion (Page 8: Lines 226-234).

6) At several places the authors write AUD GWAS, I assume they refer to the AUD GWAS meta-analysis that they perform, but this is not clear. So please correct this to AUD GWAS meta-analysis throughout the text, when relevant.

Ans: We thank the reviewer for pointing this out. We have now corrected the text as requested.

7) In the introduction the authors write DPW is genetically uncorrelated with most psychiatric disorders (except ADHD and tobacco use disorder) but correlated negatively with educational achievement and cardio-metabolic disease (which remains uncorrelated with PAU or AUD). In the GSCAN paper DPW has a non-significant genetic correlation with educational attainment of rg=0.01 (including 23andMe individuals in the DPW sample), and Kranzler et al. found a positive genetic correlation between alcohol consumption and educational attainment. So could the authors check if the sentence instead should be "but correlated positively with educational achievement".

Ans: We thank the reviewer for pointing this out, and we have corrected the sentence to "but correlated positively with educational achievement" (Page 4; Lines 95-97).

Reviewer #3 (Remarks to the Author):

The focus of this work is to integrate -omics data (expression, methylation) with genome wide association study (GWAS) results for the two outcomes of alcohol use disorder (AUD) and drinks per week (DPW) to try to understand the functional significance of variants and determine causal candidate genes. It is novel in that it is the largest multi-omics integration of these two outcomes, and will be of interest to the psychiatric and genetics research community. This is an impressive amount of work managing many different data sets and provides candidate loci of interest. The main concerns were with the stated goals of a simultaneous approach for both

outcomes, whether ancestry was addressed, and the presentation of the manuscript.

1. The introduction claims that this is an integrated approach and looks at the two phenotypes AUD and DPW "specifically as well as simultaneously". But it seems like most of the analyses examine AUD and DPW separately, then look later for overlap. For example, the LDSC analysis seems to be completely separate, and not much discussion about overlap. Can the authors do more direct analysis (e.g. SNPs that intersect for the two outcomes) for a more integrative and simultaneous approach at later stages? Or they may need to downplay this description.

> *Ans: The reviewer is correct: the analyses presented in this manuscript performed multi-omic integration analyses separately for AUD and DPW and only later looked at the overlap. We specifically employed this approach as it minimizes the bias in results due to large sample size differences between the two GWASs. The extremely large sample size for DPW GWAS means that a simultaneous multi-omics analysis would predominantly prioritize genes associated with the DPW phenotype. In fact, we used the Multi-Trait Analysis of GWAS (MTAG) method to meta-analyze the summary statistics from DPW and AUD GWASs. Although this method is robust to correlated multi-trait meta-analysis, the SMR analysis using the MTAG results were primarily driven by the DPW variants. To reduce the bias, we focused on SMR results from MTAG using our stricter threshold (GWAS AUD 5 x 10-5; e/mQTL P 5 x 10-8; SMR P < 20%; Heidi P > 0.05); the results were similar to our original SMR results (later overlap analysis). In fact, many SNPs in the combined analysis were filtered out due to inflated summary statistics of the heterogeneity test (Supplementary table 5).*
>
> *On the other hand, SMR analysis using individual summary statistics highlighted the unbiased association within each dataset. Additionally, the current discussion section highlights the strongest overlapping gene (SPI1) that passed the stricter threshold of association in DPW and AUD meta-analysis datasets (SMR FDR P < 20%, Heidi P > 0.05, GWAS P 5 x 10^{-8}, eQTL/ mQTL P < 5 x 10^{-8}). We have included an additional supplementary table (Supp table 6) that includes the overlapping summary statistics from AUD and DPW SMR results at relaxed P values. A list of all of the overlapping genes can also be visualized on the ShinyApp. The introduction section has been updated to briefly mention and justify the approach used in current study (Page 5; Lines 117-122).*

2. Ancestry is not discussed but there appears to be both European and African ancestry populations in the meta-analysis GWAS. But for the various e/mQTL meta-analysis performed by the authors and others, how similar are those populations to the populations used in the meta-analysis GWAS? Is ancestry adjusted for in any of the analyses? (GWAS, QTL, differential expression, etc)

> *Ans: For the current study we only analyzed the European subset of PGC. Genetically calculated PC1 was added in the European subset as well. We have made the changes in manuscript to better describe the analyses. The appropriate genetically calculated PCs were included in the individual analyses as well. The GWAS analyses in COGA and MVP datasets was also limited to European ancestry (Page 11, Lines 348-353).*

3. Other concerns were about the presentation regarding enough detail for reproducibility and the statistical analysis. Since space is constrained, a supplemental materials/methods would help, in addition to improved organization of the figures/tables and supplemental figures/tables.

The current analysis primarily used summary statistics from published datasets. We have included citations to the original analyses in the methods. We have also included additional details for the AUD meta-analyses, COGA-INIA eQTL analysis and eQTL meta-analysis for reproducibility. The scripts used for eQTL and SMR analysis have been added to a GitHub page (https://github.com/kapoormanav/alc_multiomics) so that others can follow and validate the results presented in the manuscript.

We have added additional descriptions/ footnotes in tables and supplementary information to improve the organization.

a. Although it may be obvious to some, it's still important to define all abbreviations (e.g, EUR, AFR, RIN). Also what is PMI? Is that supposed to be BMI?

Ans: We apologize for not expanding all the abbreviations. PMI is the post-mortem interval. We have now expanded all the abbreviations in the text.

b. What meta-analysis method is used for the "eQTL meta-analysis in adult brain" e.g., random-effects, etc. In general, for all of the meta-analyses, more detail would be important regarding the method, populations, sample sizes.

Ans: The meta-analysis for eQTL was performed using an inverse-variance-weighted meta-analysis assuming all the cohorts are independent. More details about the method can be found here (https://cnsgenomics.com/software/smr/#MeCS). The methods section has been updated for all the meta-analyses with appropriate citation to the methods.

c. The numbering of the tables/figures are off. For example, Supplementary Table 1 seems to be 2, and Figures 2 and 3 seem to be 4 and 5 respectively. Other times Supplemental Figures and Tables are referred to in the text, but with no number.

Ans: We thank the reviewer for pointing this out. We have made the required changes to cite each figure and table properly.

d. There is a file of additional supplementary figures that are not described and did not appear to be referenced. They should not be included if not cited specifically.

Ans: We thank reviewers for pointing this out. The supplementary figures are complementary to the data in the supplementary tables. It was our oversight that we didn't cite these along with these tables. We have now cited all tables and figures.

e. The manuscript states "A large proportion (45%) of AUD and DPW associated SNPs were within intronic, UTR and non-coding regions of the genome." Is this percentage expected by chance?

Ans: Compared to all variants from the reference genotyping array, the proportion of SNPs within intronic, UTR and non-coding regions was suggestively significantly enriched for AUD and DPW associated SNPs ($P < 5 \times 10^{-2}$) [Additional supplementary information figure 6].

f. Figure 6 seems unnecessary as a figure, the content can be displayed as a table or even in the text.

Ans: The content of the figure is available as supplementary information (supplementary table 4) as well. We have removed the figure according to reviewer's suggestion.

g. In the methods, please provide p-value or FDR cutoffs used in the different analyses. Is multiple testing adjustment performed for the differential expression analysis?

Ans: The methods and results section have been updated to emphasize the p-value and FDR cut-offs. The differential expression results listed in table 1b did not pass the threshold for multiple test correction due to the small sample size of the post-mortem brain dataset (N =92). The P values were listed as additional information to prioritize the candidate genes according to actual changes in gene expression due to alcohol exposure. We have added this statement under the table 1b as well.

h. Can a similar figure relating MAPT gene expression to DPW as in Figure 4C, be performed for the candidate gene SPI1 in Figure 5?

Ans: Unfortunately, we don't have access to alcohol consumption data in the myeloid cell datasets where SPI1 is primarily expressed. SPI1 expression was not in the detectable range in the bulk brain RNA-Seq data from brains of alcoholics, due to the tiny proportion of microglia in bulk brain tissues (<5%). As a result, it is not possible to create a similar figure for SPI1.

Reviewer #4 (Remarks to the Author):

This comprehensive analysis of existing human 'omics data relevant to the understanding of alcohol use disorder provides some replication of previously identified genes and evidence for novel associations. The manuscript is clearly written so it is easy to follow the different datasets

and analyses conducted. The statistical methods applied are appropriate and rigorous. It represents a significant advancement for the field of alcohol genomics and should inspire basic scientists to prioritize some of these genes for further study.

We thank the reviewer for highlighting the salient features of our work.

Some questions and suggestions:

1) Title: It is not clear to me why a reference to neurodegenerative diseases merits mention in the title. It's not irrelevant, but I don't feel the results strongly point in this direction, or at least the way presented in the paper it is not emphasized.

Ans: The top genes identified (SPI1 and MAPT) in this study are also strong candidates for Alzheimer disease risk (Huang et al, 2017, PMID: 28628103; Sanchez-Juan et al, 2019, PMID: 31866851). MAPT is also a risk gene for progressive supranuclear palsy and corticobasal degeneration and a causal gene for Fronto-temporal dementia. Additionally, the gene-enrichment analysis demonstrated strong enrichment for neuro-degenerative disease associated genes. These observations together led us to believe that there might be some connection between alcoholism and neurodegenerative disorders. Since alcoholism is more commonly compared to other substance abuse disorders and psychiatric disorders we thought it important to point out the largely overlooked overlap with neurodegenerative diseases, many of which have behavioral/personality changes as part of the spectrum of clinical symptoms.

2) Introduction, para 2: I do not understand why the authors refer to the Zhou et al paper (ref 10) as the "largest tranche of signals for any addictive disorder to date". In the very next sentence, they refer to the Liu et al paper (ref 11) which included a larger sample and identified more loci/genes.

Zhou et al. specifically focused on problematic alcohol use which is very highly correlated with alcohol use ***disorder****. Liu et al. included a larger sample size but analyzed consumption rather than disorder; the genetics of consumption and problematic alcohol use or AUD differ. We have added an additional sentence to clarify the differences between phenotypes used in the two studies (Page 4: Line 86-90).*

3) The emergence of *SP11* and *NUP160* as potentially important genes is interesting, especially given their high expression in myeloid cells. My colleagues who are experts in the connections between immunology and neurobiology have often questioned why we do not use perfusion for animal studies of mouse/rat brain for various RNA sequencing experiments. Their reasoning is that if you simply flash freeze the brains and use tissue from that, you will inevitably be assessing a lot of blood cells, not neuronal cells. Obviously, with human brains, perfusion is not possible. But this makes it difficult to interpret results where the genes involved may be more likely reflect changes in blood cells than neuronal cells. This is not to say such changes aren't

important, but it seems some discussion is necessary to help the reader hypothesize how such changes may be functionally relevant to what is being studied in this context as a "brain" disease. This is more of a "big-picture" question, because as the authors point out in the discussion, other studies have identified an important role of immune networks in drinking behaviors (ref 25-31).

Ans: Reviewer 4 makes an excellent point regarding the gene expression studies from human alcoholic brain tissue. Our current study, therefore, is important because we started with the genetic predisposition (GWAS) data and asked the question whether the disease associated variants alter gene expression in certain tissues and/ or cell types. While we cannot distinguish between brain resident cells and peripheral myeloid cells in our bulk RNAseq data from the alcoholic brains, other data point specifically to brain resident microglia. For example. using brain specific single cell epigenetic data from 4 major cell types (neurons, microglia, astrocytes and oligodendrocytes), we demonstrate that the disease associated variants specifically overlap with epigenetic signals microglia (see figure 5).

4) Minor point: Discussion, para 5 that begins, "We also identified other genes..." The word "also" occurs several times so the authors may wish to rework some of the writing.

Ans: We have now re-worded some parts of the discussion for better flow and understanding.

5) I did not see any specific discussion about whether the authors attempted to identify possible sex differences for any of the analyses. It appeared to be included as a covariate for most or all, but in some cases it seems like they may have enough power to separate the sample by sex. Given the known sex differences in AUD, this has the potential to be very interesting and would add something new to the field. While the current paper is a fantastic effort and integration, it's really just adding more genes on top of other genes from previous 'omics studies, so a sex-specific analysis would add something new.

Ans: We agree with the reviewer that it would be interesting to include sex stratified analysis. The current analyses were performed using GWAS and e/mQTL summary statistics to boost the power of the analysis. Given the limited availability of sex stratified summary statistics and complete raw data of GWAS and e/mQTL data we are not able to perform the sex stratified analysis.

Along these lines, it would be useful for the authors to identify the genes in their tables that are new with this analysis and haven't emerged from previous publications. I don't say this to imply that I don't believe they've found anything new - I agree there is lots of great new stuff here. But it would really help the reader be able to distinguish which pieces are new and which genes now have lots of evidence for involvement in AUD.

Ans: The superscript N in front of gene name in table 1a and 1b highlights the novel candidate causal genes prioritized in this study.

6) I really like the use of ShinyApp to allow others to query the database and view the results. However, it would be useful to add a section that clearly describes under a single heading where each of the datasets can be found, so that others could obtain the raw data for themselves.

We have added a GitHub link on the app where the summary statistics used in this dataset will be downloaded after the publication.

7) This paper is limited to data collected from human studies. There would value in adding a paragraph or two in the discussion about genes identified in animal models. Unfortunately, I don't think a recent GeneWeaver analysis has been published for alcohol phenotypes, but I think Rohan Palmer's group has been working on it.

Ans: We agree that data from animal studies might further add confidence to the results of the current study. We have provided evidence from animal studies in support of results of the current analyses. For example, in the discussion section we have provided evidence from animal studies in support of SPI1 as a candidate gene. Specifically, the following three references were noted in support of SPI1 as a candidate gene:

35. Socodato, R. et al. Daily alcohol intake triggers aberrant synaptic pruning leading to synapse loss and anxiety-like behavior. Sci Signal ***13****(2020).*
36. Joshi, P.C. et al. Chronic ethanol ingestion in rats decreases granulocyte-macrophage colony-stimulating factor receptor expression and downstream signaling in the alveolar macrophage. J Immunol ***175****, 6837-45 (2005).*
37. Mehta, A.J. et al. Zinc supplementation restores PU.1 and Nrf2 nuclear binding in alveolar macrophages and improves redox balance and bacterial clearance in the lungs of alcohol-fed rats. Alcohol Clin Exp Res ***35****, 1519-28 (2011).*

We have also provided evidence from animal studies in support of MAPT as a candidate gene (Hoffmann et al, 2019)
Hoffman, J.L. et al. Alcohol drinking exacerbates neural and behavioral pathology in the 3xTg-AD mouse model of Alzheimer's disease. Int Rev Neurobiol ***148****, 169-230 (2019).*

We have added an additional paragraph in the discussion to emphasize the importance of validation of human results in vitro and in-vivo. All results from the current analysis will be freely available to integrate with the gene-weaver and other cross-validation platforms as well.

Reviewers' Comments:

Reviewer #2:
Remarks to the Author:
My main concerns were related to the FDR used in the SMR analyses and lack of significance after Bonferroni correction (and their wording related to this) when testing for difference in expression of MAPT in postmortem brains. I think the authors have addressed my concerns regarding these two points and provided sufficient information regarding my other points.

I have no further comments to this manuscript.

Reviewer #3:
Remarks to the Author:
The authors have appropriately responded to my initial critiques. I have no additional comments.

Reviewer #4:
Remarks to the Author:
The authors have addressed all of my comments.
